# Hypnotic Induction of Deafness to Elementary Sounds: An Electroencephalography Case-Study and a Proposed Cognitive and Neural Scenario

**DOI:** 10.3389/fnins.2022.756651

**Published:** 2022-03-17

**Authors:** Esteban Munoz Musat, Benjamin Rohaut, Aude Sangare, Jean-Marc Benhaiem, Lionel Naccache

**Affiliations:** ^1^INSERM U1127, CNRS 7225, Paris Brain Institute, Paris, France; ^2^Sorbonne Université, Paris, France; ^3^Department of Neurology, Groupe Hospitalier Pitié-Salpêtrière, Assistance Publique–Hôpitaux de Paris, Paris, France; ^4^Department of Neurophysiology, Groupe Hospitalier Pitié-Salpêtrière, Assistance Publique–Hôpitaux de Paris, Paris, France

**Keywords:** consciousness, hypnosis, odd-ball, hypnotic deafness, global neuronal workspace theory (GNWT), top-down

## Abstract

Hypnosis can be conceived as a unique opportunity to explore how top-down effects can influence various conscious and non-conscious processes. In the field of perception, such modulatory effects have been described in distinct sensory modalities. In the present study we focused on the auditory channel and aimed at creating a radical deafness to elementary sounds by a specific hypnotic suggestion. We report here a single case-study in a highly suggestible healthy volunteer who reported a total hypnotically suggested deafness. We recorded high-density scalp EEG during an auditory odd-ball paradigm before and after hypnotic deafness suggestion. While both early auditory event-related potentials to sounds (P1) and mismatch negativity component were not affected by hypnotic deafness, we observed a total disappearance of the late P3 complex component when the subject reported being deaf. Moreover, a centro-mesial positivity was present exclusively during the hypnotic condition prior to the P3 complex. Interestingly, source localization suggested an anterior cingulate cortex (ACC) origin of this neural event. Multivariate decoding analyses confirmed and specified these findings. Resting state analyses confirmed a similar level of conscious state in both conditions, and suggested a functional disconnection between auditory areas and other cortical areas. Taken together these results suggest the following plausible scenario: (i) preserved early processing of auditory information unaffected by hypnotic suggestion, (ii) conscious setting of an inhibitory process (ACC) preventing conscious access to sounds, (iii) functional disconnection between the modular and unconscious representations of sounds and global neuronal workspace. This single subject study presents several limits that are discussed and remains open to alternative interpretations. This original proof-of-concept paves the way to a larger study that will test the predictions stemming from our theoretical model and from this first report.

## Introduction

The scientific study of consciousness has grown tremendously over the past 20 years. Initially focused on the discovery of the neural correlates of consciousness (i.e., the minimal neural mechanisms of a specific conscious experience) (Crick and Koch, 1990; Rees et al., 2002; Koch et al., 2016), it is now a maturing science with various explanatory theories that have been proposed, refined, and tested over the years (Dehaene and Naccache, 2001; Lamme, 2006; Lau, 2007; Tononi, 2012). Among these theories, the Global Neuronal Workspace Theory (GNWT; Dehaene and Naccache, 2001; Dehaene et al., 2014; Mashour et al., 2020) proposes that conscious experience arises from the late, non-linear and sustained ignition of a global network of cortical areas which allows the global broadcasting of an (initially) unconscious neural representation supported by a local cortical module. The GNWT predictions have been validated in a number of different experimental paradigms, including auditory odd-ball paradigms during wakefulness (Bekinschtein et al., 2009; Wacongne et al., 2011) or during sleep (Strauss et al., 2015); masking paradigms (Dehaene et al., 2001; Del Cul et al., 2007; Gaillard et al., 2009), attentional blink (Sergent et al., 2005) and even, more recently, in no-report paradigms (Sergent et al., 2021).

A different way of evaluating the explanatory power of consciousness theories is by testing their predictions in the face of extreme situations outside the range of normal conscious experiences, such as lucid dreaming, functional neurological disorders, states induced by psychedelics or hypnosis [a.k.a. altered states of consciousness (Ludwig, 1966)]. Hypnosis, in particular, is a special form of top-down regulation in which verbal suggestions are capable of eliciting pronounced changes in the contents of consciousness (Oakley and Halligan, 2013; Terhune et al., 2017), including (but not limited to) changes in perception, such as visual and auditory hallucinations (Spiegel, 2003; Woody and Szechtman, 2011). These hypnotic-induced perceptual changes can improve objective behavioral performance in some challenging perceptual tasks (Raz et al., 2006; Landry et al., 2021), revealing that hypnosis can genuinely change the contents of conscious perception, and not only induce a response bias.

Although hypnotic phenomena are explored by cognitive neuroscience, very few studies have explicitly tried to interpret these phenomena in the larger framework of an existing consciousness theory. Interestingly, the GNWT states that a conscious context or instruction can modulate, in a top-down manner, the unfolding of unconscious processes (Dehaene and Naccache, 2001; Naccache et al., 2002; Naccache, 2009). This prediction has been tested and confirmed in several experimental contexts such as the endogenous allocation of spatial (Kentridge et al., 2004) and temporal attention (Naccache et al., 2002) for subliminal stimuli, the conscious setting of response codes (Dehaene et al., 1998; Rohaut et al., 2016) or task-related strategies (Nakamura et al., 2007). In all these examples, once a specific “conscious posture” is adopted, it can influence unreported cognitive but also sensory and motor processes. Applied to the physiology of post-hypnotic suggestive effects, GNWT would predict a similar scenario: once a conscious and voluntary posture is adopted, it could be sustained in time and affect the corresponding cognitive or emotional processes. In the specific case of hypnotic regulation of perception, the GNWT will predict that the conscious voluntary setting of a cognitive state originating from posthypnotic suggestions will, *via* ordinary cognitive control mechanisms (involving prefrontal structures), influence neuronal workspace activity in a way that will allow the emergence of the expected perceptual experience. Hypnotic induction of a perceptual *deficit*, such as hypnotic blindness or deafness, would therefore proceed through a conscious and active cognitive inhibition process preventing the late “ignition” stage of processing associated with the global broadcasting of the sensory information, resulting in the subjective experience of blindness or deafness. This former inhibition process will likely involve dorsal anterior cingulate cortex (dACC) activation, since this cortical structure is essential in conflict monitoring and inhibitory control (van Veen and Carter, 2002).

In the present study, we report the case of a hypnotic suggested deafness to elementary sounds in a highly suggestible individual. We studied resting state activity and brain responses to sounds using high-density electroencephalography (EEG) during a classical auditory odd-ball paradigm (Squires et al., 1975), before and after hypnotic suggestion of deafness. To the best of our knowledge, only one study has investigated before hypnotic deafness using an odd-ball paradigm (Franz et al., 2020). Using a combination of evoked related potentials, source localization, machine learning decoding methods and resting state markers, we make the exposed main predictions of the GNWT very plausible in this case of hypnotic deafness, by showing during the post-hypnotic suggestion period: (i) a preservation of early unconscious processing, with unchanged cortical responses to sounds and mismatch negativity component; (ii) the existence of an active inhibitory process probably mediated by the dACC; (iii) the blockade of the non-linear ignition process associated with conscious access, as revealed by the blockade of the late P3b component; (iv) a functional disconnection between the modular and unconscious representations of sounds and global neuronal workspace.

## Materials and Methods

### Participant

This study reports the case of one right-handed 24 years old female healthy participant with no neurological or psychiatric medical history, with a high hypnotic suggestibility profile assessed by a certified physician with expert level training in hypnosis (Head of the Sorbonne University Medical Hypnosis University Diploma) and scored with the French adaptation of the shortened version of forms A and C of the Stanford Hypnotic Susceptibility Scale (Raynaud et al., 1984). The participant was referred to us by her hypnotherapist because of her high suggestibility profile and her willingness to help in the scientific understanding of hypnotic phenomena. The volunteer gave her informed consent to participate to this study. This experiment was approved by the Ethical Committee of the Kremlin-Bicêtre Hospital (no. 98-25).

### Experimental Design

**FIGURE 1:**
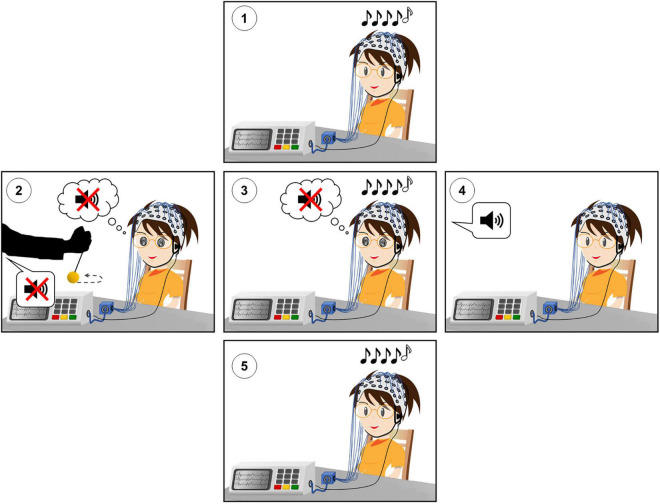
Experimental design of the hypnotic deafness paradigm. (1) Baseline (PRE) session of the auditory odd-ball paradigm. (2) Hypnotic induction and suggestion of radical deafness to elementary sounds. (3) Hypnotically suggested deafness (HYP) session of the auditory odd-ball paradigm. (4) Termination of hypnosis and recovery of subjective experience of sound. (5) Post-hypnotic (POST) session of the auditory odd-ball paradigm. During each session of the auditory odd-ball paradigm, series of five sounds were presented, with the last sound being identical (AAAAA or BBBBB, “standard”) or different (AAAAB or BBBBA, “deviant”) than the first four. High-density scalp EEG was recorded during each experimental session.

The experimental design is shown in Figure 1.

We first recorded high-density EEG (HD-EEG) during a classical auditory odd-ball paradigm [(Squires et al., 1975), see below] in a passive attentive condition [see for instance experiment 2 of Quirins et al. (2018)]. This baseline session hereafter referred to as the PRE condition, was then followed by a hypnotic procedure with the suggestion of deafness to sounds. We then recorded again HD-EEG during the same odd-ball paradigm (HYP condition). After this HYP session, the hypnosis state was terminated and the volunteer fully recovered her conscious auditory perception. Finally, she was recorded a third and last time with the odd-ball paradigm (POST condition). During the three experimental sessions of the odd-ball paradigm, the volunteer kept her eyes closed and was instructed to simply attend to the sounds without any other task (passive attentive state).

The hypnotically suggested deafness to elementary sounds was performed by a certified hypnotherapist physician (JMB). After a standard induction procedure, the suggestion of being deaf to elementary sounds or noises (but remaining aware of voices) was delivered. Then, the volunteer was stimulated with one series of five identical sounds used for the odd-ball paradigm, and she had to report through a motor code instructed before the induction if she perceived consciously these sounds. The suggestion procedure continued until the volunteer reported the absence of conscious perception of sounds. After two first attempts during which she reported a conscious perception of sounds, she reported subjective deafness to sounds at the third attempt. The total duration of the induction/suggestion procedure was 14 min.

### Auditory Odd-Ball Paradigm

Series of five complex 50-ms-duration sounds were presented *via* headphones with an intensity of 70 dB and 150 ms Stimulus Onset Asynchrony (SOA) between sounds. Each sound was composed of three sinusoidal tones (either 350, 700, and 1,400 Hz, hereafter sound A; or 500, 1,000, and 2,000 Hz, hereafter sound B). All tones were prepared with 7-ms rise and 7-ms fall times. Four different series of sounds were used, the first two using the same five sounds (AAAAA or BBBBB); and the second with the final sound swapped (either AAAAB or BBBBA). Series of sounds were separated by a variable interval of 1,350–1,650 ms (50-ms steps). For the two conditions (PRE and HYP), the participant heard two stimulus blocks, block type a: 67% AAAAA/33% AAAAB; and block type b: 67% BBBBB/33% BBBBA. Each block contained 78 series of sounds, for a duration of about 4 min. Series of sounds AAAAA and BBBBB are hereafter called standard stimuli, and series AAAAB and BBBBA deviant stimuli (since the fifth sound of the series is a deviant compared to the first four sounds).

### High-Density Scalp Electroencephalography Acquisition and Preprocessing

High-density scalp EEG were acquired using 256 electrodes Hydrocel Geodesic Sensor Net on a Net300 Amplifier (Electrical Geodesic, Eugene, OR, United States) with a sample frequency of 250 Hz. Impedances were set to below 75 kΩ prior the start of the first recording.

Raw EEG files were band-pass filtered between 0.5 and 10 Hz for event related potentials (ERPs) and decoding analysis, and between 0.5 and 45 Hz for spectral power and functional connectivity analysis, with 50 and 100 Hz notch filters.

Trials were then segmented from −200 to +1,400 ms relative to the onset of the first sound. The obtained epochs were then cleaned, based on their voltage maximum amplitude and variability, using a fully automatized procedure previously published (Engemann et al., 2015). More precisely, channels that exceeded a 100 μV peak-to-peak amplitude in more than 50% of the epochs were rejected. Channels that exceeded a z-score of 4 across all the channels mean variance were rejected. This step was repeated two times. Epochs that exceeded a 100 μv peak-to-peak amplitude in more than 10% of the channels were rejected. Rejected channels were interpolated. EEG were deemed to pass this preprocessing step if at least 70% of the channels and at least 70% of the epochs were kept.

The remaining epochs were digitally transformed to an average reference, realigned relative to the onset of the fifth sound (−800 to +800 ms) and then corrected for baseline over the 800 ms window prior to the fifth-sound onset. For spectral power and functional connectivity analysis, we applied a baseline correction over the 200 ms window prior to the onset of the first sound (−800 to −600 ms relative to the onset of the fifth sound).

For the PRE and HYP conditions, trial rejection rates were low (inferior to 8%). However, note that the long duration of the hypnosis session caused electrodes to progressively dry and electrodes impedances to increase. As a consequence, the POST session was rejected due to systematic artifact rejection on most of the trials.

### Event-Related Potentials Analysis

#### Sensor Space Analysis

##### Event Related Potential Components

In this auditory odd-ball paradigm, we investigated the classical ERPs known to be evoked by deviant stimuli, namely the mismatch negativity (MMN), the P3a component and the P3b component. We also investigated the P1 evoked response of the first sound. Each ERP component was studied in a predefined spatial region of interest (ROI) and predefined time-window, according to previously published work (Bekinschtein et al., 2009; Sitt et al., 2014). Three spatial ROIs were used: one for the P1 component; one for the MMN and P3a components; and finally, one for the P3b component (for a list of the sensors comprising the different ROIs, see Sitt et al., 2014, Supplementary Material). The predefined time-windows, relative to the onset of the fifth sound, were as follows: for the P1 component, from −532 to −484 ms; for the MMN, from 140 to 192 ms; for the P3a, from 280 to 340 ms; for the P3b, from 400 to 800 ms.

##### Statistical Analysis

We studied in a frequentist approach the effects on brain responses of two main factors and their interaction: the condition or STATE (PRE vs. HYP) and the STIMULUS type (standard vs. deviant). To probe the effect of hypnotic deafness on brain responses to deviant sounds, we were particularly interested in the interaction of the two main effects (STATE × STIMULUS).

We first conducted an analysis on each of our predefined spatial ROI, by averaging the signals of the sensors constitutive of each ROI. We conducted a mass-univariate analysis at each time point, using a 2 way-type II ANOVA with STATE and STIMULUS as between-trials explanatory factors. We corrected for multiple tests using a Benjamini–Hochberg False Discovery Rate (FDR) procedure, with an alpha level of 0.05 (Benjamini and Hochberg, 1995).

We next performed an analysis averaging each spatial ROI signal within its corresponding time-window. For each component (P1, MMN, P3a, and P3b), we computed its average amplitude, as the average of voltage in its predefined time-window, over its predefined spatial ROI. We performed a similar frequentist 2-way ANOVA analysis than before (with STATE and STIMULUS as between-trials explanatory factors), with pairwise *t*-tests as *post hoc* analysis (FDR corrected for multiple comparisons). For effect size estimation, we computed partial eta squared (η^2^) for ANOVA analyses, and Cohen’s *d* for pairwise *t*-tests. For further exploration of null results, we also computed Bayesian ANOVA analyses with the same factors as before (STATE and STIMULUS), as well as Bayesian *t*-tests for pairwise comparisons [we computed the Bayes Factor (BF) H1/H0, with H1 being the “true hypothesis” (existence of a difference between the two conditions) and H0 being the “null hypothesis” (the two conditions are equal)].

#### Source Space Analysis

##### Forward Model and Source Modeling

We used a constrained distributed model consisting in 15,000 current dipoles, constrained to the cortical mantle of a generic brain model obtained from the BrainStorm software package^1^. EEG forward modeling was computed using a symmetric Boundary Element Method (BEM) head model. Current density maps were computed using a minimum norm imaging method (Baillet et al., 2001).

##### Source Estimation of the Odd-Ball Effect

We computed the evoked odd-ball effect as the subtraction of the average of deviant trials minus the average of standard trials, separately for each state (PRE and HYP, respectively) and modeled the corresponding sources. To probe the significance of source activity, we performed at each time point a *t*-test against the baseline (−800 to 0 ms), and corrected for multiple tests in the spatial and temporal dimensions using a FDR procedure, with an alpha level of 0.001.

##### Statistical Analysis of Source Activity at Dorsal Anterior Cingulate Cortex

We extracted trial-to-trial source activity time series of right and left dorsal anterior cingulate cortex (dACC), using a standard anatomical atlas (Desikan-Killiany, Desikan et al., 2006). We then averaged the signal in the predefined P3a time window (280–340 ms), and performed a similar frequentist analysis as described for sensor space, with STATE and STIMULUS as between-trials factors. As before, we added to this frequentist analysis a Bayesian analysis.

### Decoding of Brain Activity

We implemented multivariate pattern classifiers (MVPA), using a linear kernel support vector machine (SVM), to further test the differences in brain evoked activity between standard and deviant trials, for the two states/conditions (PRE and HYP).

#### Temporal Decoding

We trained a multivariate predictive model on each time instant to distinguish between standard and deviant trials. We trained two sets of classifiers: one with the PRE data, and one with the HYP data. For each time point, the amplitude of each electrode was provided to the classifier and normalized across training trials. The SVM was fitted to find the linear hyperplane that best separates standard from deviant trials and a cumulative probability distribution function was then fitted to the training set using Platt’s method (Platt, 1999). To take into account data unbalance (more standard trials than deviant ones), the weights of each class were adjusted in an inversely proportional manner to class frequencies.

We then evaluated the performance of the classifiers at each time instant, both for the PRE and the HYP data. Classification scores for each time instant were estimated from the predicted probabilities of the trials from the testing sets and summarized with the area under the curve (AUC).

To avoid over-fitting and circular analysis, when the training data was the same as the test data (ex: testing PRE data with classifiers trained with PRE epochs), a standard seven-fold stratified cross-validation procedure was implemented for the training and testing steps.

#### Temporal Generalization Decoding

Temporal generalization is an extension of the decoding over time approach. It consists of evaluating whether the model estimated at a particular time instant accurately predicts any other time instant (King and Dehaene, 2014). This is repeated for all possible training time-points to create a full matrix of accuracy for every combination of train/test time-points. This method can show if brain activity patterns are transient or sustained, thus allowing us to track neural representations over time. For each state, we trained and tested a set of classifiers according to this principle, using a five-fold stratified cross-validation procedure. The same machine learning methods used for the standard temporal decoding were applied for the temporal generalization decoding.

#### Statistical Analysis

We tested the significance against chance of the obtained AUC using a 500 permutation procedure, at each time point. More specifically, at each time-point, and for each permutation, trial labels were randomly shuffled and the whole decoding procedure was repeated, thus allowing us at the end of the permutation procedure to obtain a distribution of surrogate AUC for each time point. These surrogate distributions were used to compute the (uncorrected) *p* value at each time-point, by counting the number of permutation scores equal or higher to the true AUC, and dividing by the number of permutations plus one. We then corrected the obtained *p* values for multiple tests using a FDR procedure, at alpha level 0.05.

### Spectral Power and Functional Connectivity Analysis

#### Spectral Power and Functional Connectivity Quantification

Additionally, we investigated the effects of the hypnotic deafness state on power spectral densities (PSD) and connectivity measures computed during the 800 ms time-window before the onset of the fifth sound (pseudo-resting state) as previously reported (Sitt et al., 2014; Engemann et al., 2018). We computed normalized PSD in delta (1–4 Hz), theta (4–8 Hz), alpha (8–12 Hz), beta (12–30 Hz), and gamma band (30–45 Hz) and the weighted symbolic mutual information (wSMI), a functional connectivity measure capturing linear and non-linear coupling between sensors which relies on the symbolic transformation of EEG signal, in the delta, theta and alpha bands (King et al., 2013). All these measures were computed for each scalp sensor, and for each epoch. Scalp topographies of wSMI measures were obtained by computing the median connectivity of each sensor with all other sensors.

#### Statistical Analysis

We then compared the obtained PRE and HYP distributions of PSD and wSMI scalp topographies by using independent *t*-tests at each one of the 256 scalp sensors, followed by a cluster-based permutation test using the cluster mass metric to control for multiple comparisons with 10,000 random permutations. Additionally, we computed for each marker and at each sensor the Bayes Factor (BF) of the corresponding comparison (H1: “the mean value of the marker is different for PRE and HYP conditions,” H0: null hypothesis, “the mean value is identical for PRE and HYP”).

### Software

All sensor space analysis (ERPs, decoding, spectral, and connectivity measures) were performed with custom scripts using Python (version 3.7.1) with MNE-python (Gramfort et al., 2013) and with scipy and pinguoin packages (Vallat, 2018) for statistical analysis. Bayesian ANOVA analyses were performed using JASP^2^.

Source space reconstruction and massive univariate analysis in source space were performed using the BrainStorm software package (see text footnote 1).

## Results

The high hypnotic suggestibility profile of the volunteer was confirmed by a maximal scoring of 8/8 (Raynaud et al., 1984) assessed before the hypnotic induction. Prior to the induction we instructed her to communicate her subjective report to us through a motor code in order to prevent vocalization of her own voice. Subjectively, the volunteer reported a conscious experience of total deafness to delivered sounds, that occurred precisely after 14 min of verbal induction (see section “Materials and Methods”).

### Significant Modulation of Late Event Related Potential Responses (P3a, P3b) by the Hypnotic Suggestion of Deafness

**FIGURE 2:**
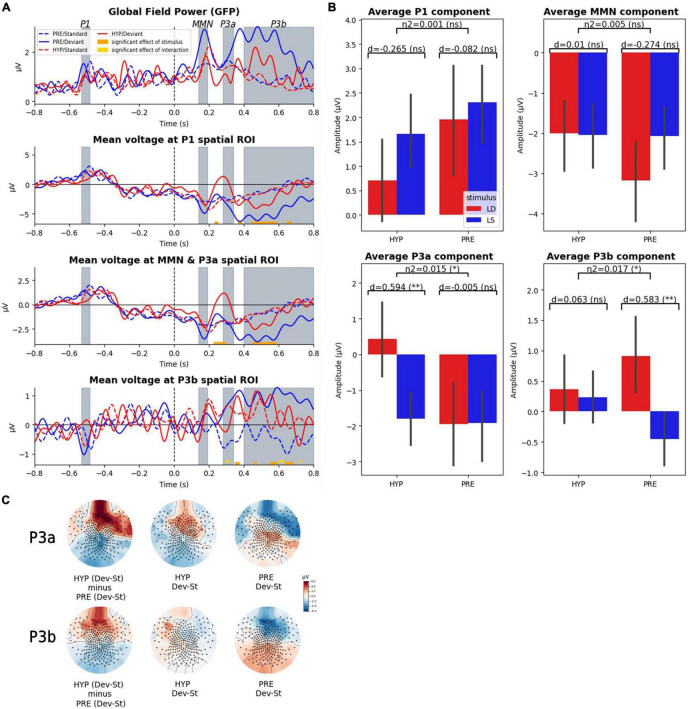
Late ERP components (P3a, P3b) were affected by hypnotic deafness while early components were not (P1, MMN). Scalp evoked-related potentials (ERPs) during the auditory odd-ball paradigm, for baseline (PRE) and hypnotic deafness (HYP) conditions. **(A)** Time-series of the global field power (GFP) and the ERPs in three predefined spatial regions of interest. Doted lines show ERPs for standard stimuli and complete lines for deviant stimuli, for PRE condition (blue) and HYP condition (red). Bottom color lines show the time intervals that are statistically significant (FDR corrected) for the main effect of STIMULUS (orange) or the INTERACTION (gold) between STATE and STIMULUS. Gray-colored intervals correspond to the predefined time-windows of the P1, the MMN, the P3a, and the P3b components. **(B)** Average ERP components (P1, MMN, P3a, P3b) for standard (LS, blue) and deviant (LD, red) stimuli, in function of state (PRE and HYP). The plot shows the interaction effect of state × stimulus and the pairwise comparison of standard and deviant stimuli for each state. We found an increased P3a response (deviant minus standard) exclusively during HYP, and the presence of a significant P3b response (deviant minus standard) exclusively during PRE. **(C)** Topographical maps of the evoked odd-ball responses (deviant minus standard) in the predefined time-windows of the P3a and the P3b components for the PRE condition (right), the HYP condition (middle) and their subtraction (HYP minus PRE, left pannel). Note the absence of the canonical centro-posterior positivity during the P3b time-window in the HYP condition, but also the apparition of a centro-anterior positivity in the P3a time-window. ns: non-significant (*p*-value > 0.05); * FDR corrected *p*-value < 0.05; ** FDR corrected *p*-value < 0.01.

We first examined how ERPs were affected by this hypnotic suggestion associated with a subjective report of conscious deafness to sounds. We performed a 2-way ANOVA with STATE (PRE vs. HYP) and STIMULUS (standard vs. deviant) as factors at each time-point for each predefined spatial ROI (P1, MMN, and P3a, P3b; see section “Materials and Methods” for details and Figure 2A). While we did not observe any significant main effect of STATE over the whole period, we identified a significant main effect of STIMULUS in each of the three spatial ROIs. As a control we could confirm the absence of STIMULUS effect prior to the onset of the fifth sound, and in particular in the predefined P1 ROI and time-window. The first STIMULUS effect was observed in the P1 spatial ROI from 232 to 252 ms. We then identified STIMULUS effects in the MMN and P3a spatial ROI (from 228 to 300 ms and from 448 to 584 ms), in the P1 spatial ROI (368–388 ms, 444–588 ms, and 644–672 ms) and in the P3b spatial ROI (348–372 ms, 460–472 ms, 548–588 ms, and 636–680 ms).

Crucially and as we predicted, a significant interaction between STATE and STIMULUS factors was found exclusively over the P3b spatial ROI in the late time-window associated with the P3 components. More precisely, we identified three significant temporal clusters (296–316 ms, 600–628 ms, and 712–724 ms). For each of these clusters we observed a larger difference between deviant and standard trials in the PRE state than in the HYP state.

Restricted analysis on the spatio-temporal locations of our ERP components of interest (P1, MMN, P3a, P3b) confirmed our previous results: we found no significant interaction effect of STATE × STIMULUS on the mean amplitude of P1 and MMN components, but we did find a significant interaction effect on the mean amplitude of the P3a and P3b components [for the mean P3a: partial η^2^ = 0.015, *F*(1) = 4.52, *p*-value = 0.034; for the mean P3b: partial η^2^ = 0.017, *F*(1) = 5.16, *p*-value = 0.024] (see Figure 2B). *Post hoc* analysis confirmed a significant P3b response (deviant minus standard) in the PRE state [1.36 μV amplitude; pairwise *t*-test: *d* = 0.58, *T*(101.27) = 3.42, corrected *p*-value = 0.0018], while we did not observe any significant P3b in the HYP condition [0.13 μV; pairwise *t*-test: *d* = 0.06, *T*(104.22) = 0.36, corrected *p*-value = 0.72]. The P3b response in the PRE condition had the canonical centro-posterior topography that has been described elsewhere (Sergent et al., 2005; Del Cul et al., 2007; Bekinschtein et al., 2009), while this topography was not observed in the HYP condition (Figure 2C). Interestingly, we discovered a higher amplitude of the mean P3a in the HYP condition [2.23 μV; pairwise *t*-test: *d* = 0.59, *T*(99.61) = 3.38, corrected *p*-value = 0.002], whereas no such effect could be observed in the PRE condition [0.02 μV; pairwise *t*-test: *d* = −0.005, *T*(127.84) = −0.03, corrected *p*-value = 0.97] (Figure 2B). This increased P3a like component had a centro-anterior topography (Figure 2C).

In order to further explore our null results, we decided to conduct supplementary Bayesian analyses to search for evidence for the null hypotheses (real absence of an effect). We started by conducting a Bayesian ANOVA over the components for which we didn’t find a significant interaction effect (STATE × STIMULUS) in frequentist analysis. These analyses confirmed the absence of a modulation of the P1 component and the MMN by the hypnotic suggestion of deafness, with moderate to strong evidence (except for the main effect of the STATE for the P1, for which the evidence was anecdotal): for the P1 component, BF10 = 0.044 (equal to BF01 = 22.7) for the interaction term state × stimulus and BF10 = 0.69 for the main effect of state; for the MMN, BF10 = 0.022 (equal to BF01 = 45.5) for the interaction term, and BF10 = 0.190 (equal to BF01 = 5.26) for the main effect of state. We next decided to investigate our main null findings: the absence of a significant P3b response in the HYP condition, but also the absence of the anomalous P3a response (observed in HYP) in the PRE condition. We found moderate evidence in favor of the absence (null hypothesis) of a P3b response in the HYP condition (BF10 = 0.2, equal to BF01 = 5), while in contrast we found very strong evidence in favor of the presence of a P3b response in the PRE condition (BF10 = 34). Moreover, we found strong evidence in favor of an increased P3a response in the HYP condition (BF10 = 29.9), while in contrast there was moderate evidence in favor of the absence of this effect in the PRE condition (BF10 = 0.18 equal to BF01 = 5.6).

As an interim conclusion, these first results confirmed our prediction of an absence of the late P3b response in the HYP condition, and further pointed to the P3a-like component as a possible locus of gating mechanism preventing access of auditory representations to a late conscious stage. Indeed, this P3a-like component was detected only in the HYP condition.

### Absence of Late Auditory “Odd-Ball” Effect Within Prefrontal Areas During Hypnotic Deafness

To further explore these results, and in particular the cortical origin of this increased P3a response for deviant stimuli during hypnosis, we performed source modeling of the mean evoked odd-ball effect, i.e., the average of deviant trials minus the average of standard trials, for each state (PRE and HYP). We then explored for each state the statistical significance of the obtained source activities by performing a *t*-test against the 800 ms baseline before the onset of the fifth sound.

**FIGURE 3:**
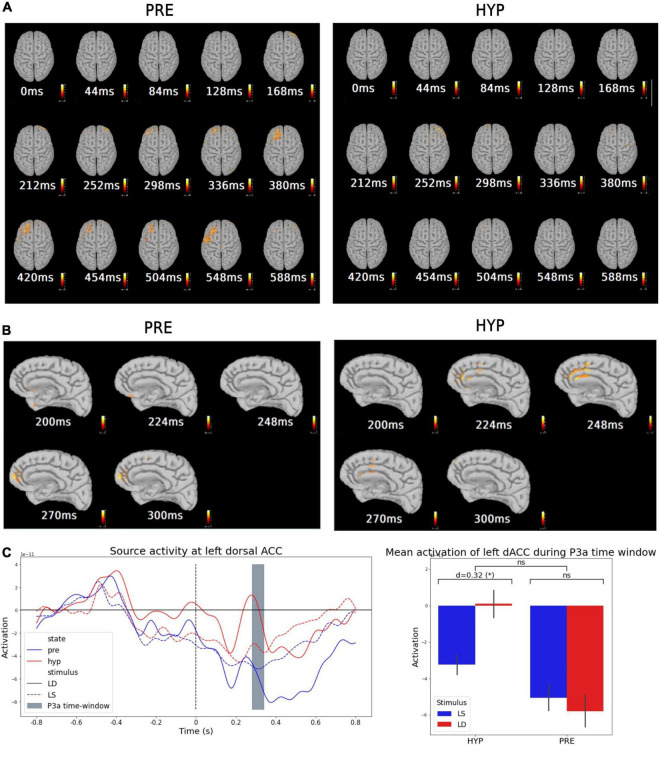
Source modeling suggested an inhibitory activation of medial prefrontal structures (dACC) during hypnotic deafness preventing activation of dorso-lateral prefrontal areas. Source modeling of the evoked-related potentials during baseline (PRE) and hypnotic deafness (HYP) conditions. **(A,B)** Sources of the mean evoked odd-ball effect (average of deviant trials minus average of standard trials) for PRE (left) and HYP (right) conditions [*t*-test against the 800 ms baseline before the onset of the 5th sound, FDR corrected in time and space dimensions (alpha = 0.001)]. While a dorso-lateral prefrontal activation was observed from 298 to 548 ms in the PRE condition (previously associated with conscious access), this activation was not observed in the HYP condition. **(B)** Presents a right-medial sagittal view during the 200–300 ms time-window, where a transient activation of medial prefrontal structures (and in particular dorsal anterior cingulate cortex) was observed exclusively in the HYP condition. **(C)** Source activity at left dorsal anterior cingulate cortex (dACC). Left panel: Time-series of the average source activity in the left dACC, in function of stimulus [standard (LS): dotted lines; deviant (LD): complete lines] and state (PRE: blue; HYP: red). The predefined P3a time-window is colored in gray. Right panel: Mean activation of left dACC during the P3a time-window, for standard (LS, blue) and deviant (LD, red) stimuli, in function of state (PRE and HYP). The plot shows the interaction effect of state × stimulus and the pairwise comparison of standard and deviant stimuli for each state. Greater activation of left dACC was observed for deviant trials exclusively in the HYP condition. ns: non-significant (*p*-value > 0.05); *p-value < 0.05.

In the PRE condition, we found a significant sustained activation in the 300–600 ms time-window, previously reported in conscious access literature (e.g., Sergent et al., 2005; Del Cul et al., 2007; Bekinschtein et al., 2009; Salti et al., 2012), involving mainly frontal lobe areas, and corresponding to the cortical sources of the P300 component (P3a and P3b) (see Figure 3A, left). As expected from our previous results and our guiding hypothesis, this late and sustained frontal lobe activation almost completely disappeared during the HYP condition (Figure 3A, right). Odd-ball related source activation in the HYP condition was almost limited to a transient activation in the 200–300 ms time-window.

### An Anterior Cingulate Cortex Mediated Inhibitory Effect Preventing Conscious Access?

Further exploration of this 200–300 ms time-window showed different source activation between HYP and PRE conditions. In particular, we found a significant activation of dorso-medial prefrontal structures in this time-window in the HYP condition, involving mainly what appeared to be the dorsal anterior cingulate cortex (dorsal ACC) (Figure 3B, right). This significant activation was not observed in the PRE condition (Figure 3B, left).

In view of these results, we wanted to test the hypothesis that the increased P3a response for deviant stimuli in the HYP condition was linked to dorsal ACC activation during the 200–300 ms time-window. We thus decided to extract trial-to-trial source activity time series of right and left dorsal ACC using a standard anatomical atlas (Desikan-Killiany) (Figure 3C, left). We averaged the signal in the predefined P3a time-window, and performed a 2-way ANOVA with STATE and STIMULUS as between-trials explanatory factors. We did not find a significant STATE × STIMULUS interaction neither in the left nor the right dorsal ACC [right: *F*(1) = 1.17, *p*-value = 0.28; left: *F*(1) = 1.66, *p*-value = 0.19] (Figure 3C, right plot) nor a main effect of the STIMULUS [right: *F*(1) = 0.5, *p*-value = 0.48; left: *F*(1) = 0.58, *p*-value = 0.45]. However, we found a main effect of the STATE in dorsal ACC activity in the P3a time-window [right: partial η^2^ = 0.016, *F*(1) = 4.7, *p*-value = 0.031; left: partial η^2^ = 0.016, *F*(1) = 4.55, *p*-value = 0.034]. Interestingly, *post hoc* pairwise *t*-tests confirmed a higher dorsal ACC activation for the HYP condition compared to the PRE condition [right: *d* = 0.26, *T*(282.1) = 2.22, *p*-value = 0.014; left: *d* = 0.25, *T*(277.9) = 2.19, *p*-value = 0.015], but also revealed a significant higher dorsal ACC activation for deviant trials compared to standard ones exclusively in the HYP condition. This last result was significant in the left dorsal ACC, and presented a clear statistical trend in the right dorsal ACC [for left dorsal ACC: HYP deviant vs. standard: *d* = 0.32, *T*(97.5) = 1.79, *p*-value = 0.038; PRE deviant vs. standard: *d* = −0.05, *T*(112.7) = −0.31, *p*-value = 0.62, BF10 = 0.38; for right dorsal ACC: HYP deviant vs. standard: *d* = 0.27, *T*(101.1) = 1.53, *p*-value = 0.065; PRE deviant vs. standard: *d* = −0.04, *T*(113.9) = −0.22, *p*-value = 0.59, BF10 = 0.38] (Figure 3C, right plot).

### Impossibility to Decode Auditory Stimuli During Hypnotic Deafness

**FIGURE 4:**
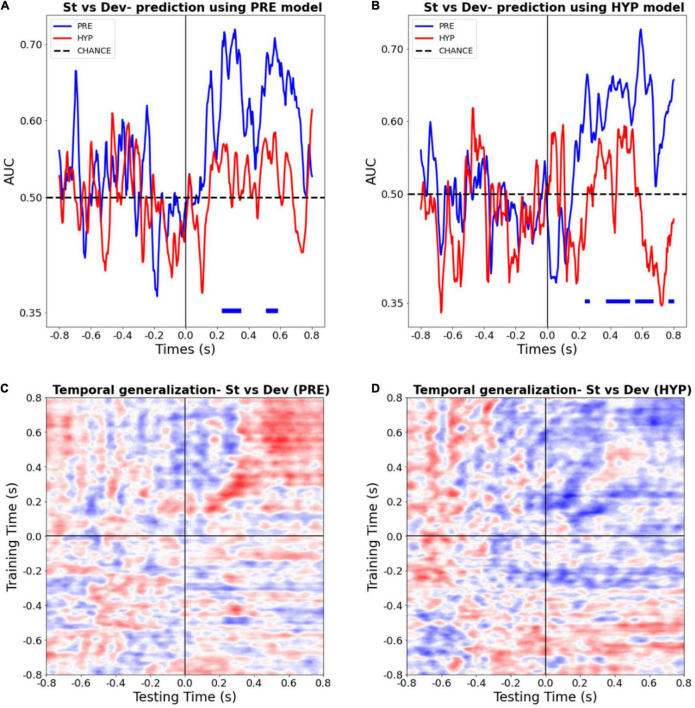
Multivariate pattern classifiers failed to decode stimulus type (deviant vs. standard) during hypnotic deafness. **(A,B)** Temporal decoding. Performance (AUC) in function of time of a classifier trained to distinguish between standard (St) and deviant (Dev) trials, tested in PRE (blue) and HYP (red) conditions. The classifier was trained either with PRE **(A)** or HYP **(B)** data, and then tested in both conditions. Bottom color lines show the time intervals where the AUC was statistically significant (FDR corrected) against chance (500 permutation procedure). While above chance decoding was observed for the PRE condition, in particular in the P300 time-window, this was not the case for the HYP condition. **(C,D)** Temporal generalization decoding procedure. The performance (AUC) of a classifier trained to distinguish between standard and deviant trials at a given time-point was tested at all other time points, for PRE **(C)** and HYP **(D)** conditions. For PRE, a pattern already described in the literature (King and Dehaene, 2014) was observed, with an early “diagonal” shape of the decoding, suggestive of a series of transient, unconscious stages of processing, and a late “square-shaped” pattern of the decoding, suggestive of a sustained stage of processing, previously associated with conscious access. For HYP, while the first component was preserved, we didn’t observe the late square-shaped component of the decoding pattern, suggesting a specific abolishment of the late conscious stage of processing.

To further explore the power of the conscious deafness induced by hypnosis, we trained a multivariate predictive model to distinguish between standard and deviant trials, in each state, for each time point. We first trained two sets of classifiers (one with PRE data and one with HYP data), and tested their classification performance on the corresponding data, using a standard stratified cross-validation procedure (Figures 4A,B), at each time point. We then tested the statistical significance against chance of the obtained AUC, using a permutation procedure, and corrected for multiple tests in the temporal dimension using a FDR procedure. In line with our previous results, we found a significant decoding during the PRE condition, on time-intervals 228–348 ms (AUC max = 0.72) and 508–580 ms (AUC max = 0.71) (FDR corrected *p*-value < 0.05). By contrast, we found no statistically significant decoding during the HYP condition (AUC max over the whole period = 0.62 min, *p*-value = 0.99).

In order to check that the absence of significant decoding during the HYP condition could not be explained by a trivial signal-to-noise ratio issue that, – if present –, should have compromised the training procedure of the decoding analysis, we tested the HYP data using the PRE training model, and vice-versa. Crucially, PRE data was still decoded above chance using the HYP model, whereas HYP data wasn’t significantly decoded using the PRE model (Figures 4A,B).

Finally, in order to better estimate the formats and durations of auditory novelty representations present during the PRE and the HYP sessions, we ran a time-generalization decoding procedure. As previously reported (King and Dehaene, 2014), the decoding pattern during the PRE condition presented two successive shapes. Its first component (∼150–400 ms) presented a diagonal-shape (see Figure 4C) reflecting a “ballistic” series of successive and transient decoding stages, without sustained time-generalization. In sharp contrast the later decoding pattern (400–800 ms) presented the typical square-like shape indicative of a sustained pattern of decoding over time. Of special interest, several studies pointed (King and Dehaene, 2014; Sanchez et al., 2020) to this second pattern as a signature of consciously accessed perceptual representations. During the HYP condition, this time-generalization method revealed a preservation of the first component of the decoding pattern (from ∼150 to 400 ms), but an absence of the second component (see Figure 4D). This last result was in favor of a specific abolishment of the late conscious stage of processing in the HYP condition.

### Significant Modulation of State Markers by Hypnosis

**FIGURE 5:**
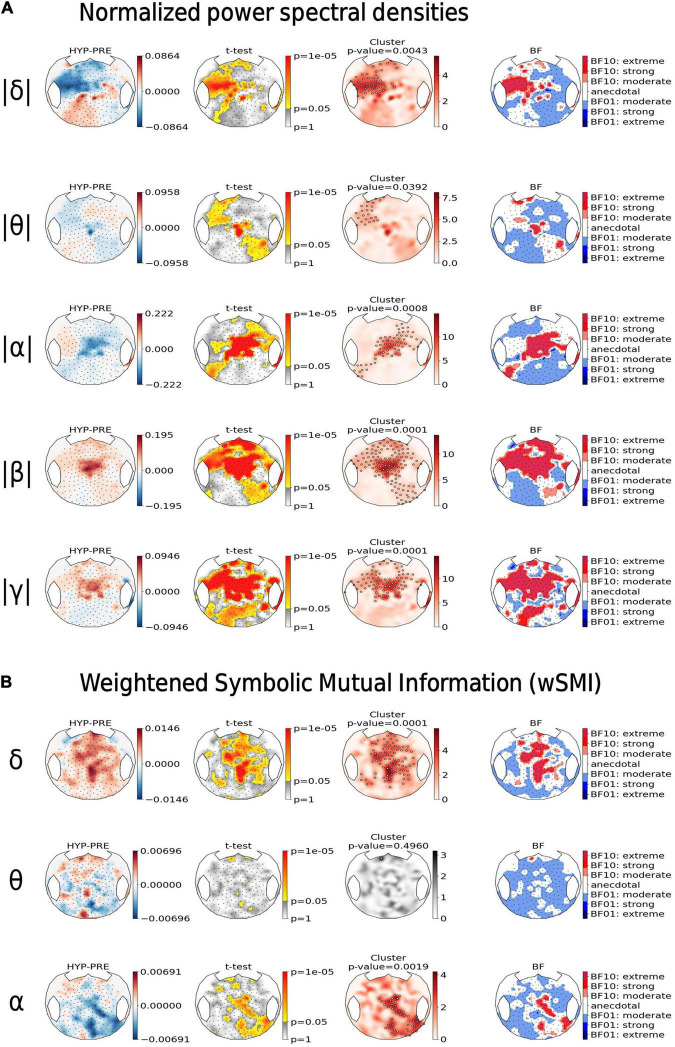
Hypnotic deafness was associated with an increase in high-frequencies power and band-specific modulation of functional connectivity. Topographical maps of the normalized power spectral densities (PSD) **(A)** and the connectivity marker *weighted symbolic mutual information* (wSMI) **(B)**, computed at different frequency bands [delta (δ), theta (θ), alpha (α), beta (β), and gamma (γ)]. For each state marker (row), from left to right: the first plot shows the contrast between baseline (PRE) and hypnotic deafness (HYP) states (HYP minus PRE), at each sensor; the second plot shows the result (uncorrected *p*-value in a logarithmic scale) of the independent *t*-test (HYP vs. PRE), at each sensor; the third plot shows the results of a cluster-based permutation test for control of multiple comparisons (red: significant cluster; gray: non-significant cluster); and the fourth plot shows the Bayes Factor (logarithmic scale) of the corresponding *t*-test (BF10: in favor of the existence of a difference between the two conditions; BF01: in favor of the null-hypothesis). Compared to baseline condition, hypnotic deafness was associated with a decrease in low-frequencies power over a left-anterior cluster, an increase in high-frequencies power over a centro-anterior cluster and band specific changes in functional connectivity (increase in delta band, decrease in alpha band, and absence of significant modification in the theta band).

Finally, we decided to further describe PRE and HYP conditions by comparing EEG state markers that were previously designed and used to describe various conscious and non-conscious states. We focused on various spectral power and functional connectivity measures. We therefore computed in different frequency bands both scalp PSDs and the wSMI that is a powerful functional connectivity measure. Importantly, these state markers were computed in the 800 ms time-window preceding the onset of the fifth sound, as in previously published work (King et al., 2013; Sitt et al., 2014; Engemann et al., 2018). We then compared the obtained scalp topographies of each of these markers between the PRE and the HYP conditions (Figure 5).

Compared to baseline condition (PRE), the hypnotic deafness condition (HYP) was associated with a significant modulation of PSD in all frequency bands. A left-anterior cluster showed a decrease of normalized PSDs during HYP as compared to PRE both in the delta band (cluster *p*-value = 0.0043) and in the theta band (cluster *p*-value = 0.039). Moreover, centro-anterior electrodes normalized PSDs decreased in the alpha band (cluster *p*-value = 0.0008), and increased in the faster beta (cluster *p*-value = 0.0001) and gamma (cluster *p*-value = 0.0001) bands (see Figure 5, top). BF topographical analysis was in favor of an extreme level of evidence for most of the above exposed results (with the exception of the left-anterior cluster for theta PSD, for which the evidence was mostly anecdotal).

Concerning this modulation of spectral power by our hypnotic procedure, it should be noted that our results only reflect the overall changes of spectral power values between PRE and HYP, without enabling us to determine the directionality of these effects in terms of increase/decrease compared to baseline level free of any sounds. To answer to this specific question of directionality compared to baseline, we should first compute spectral power values in the corresponding baselines free of any sounds (−200 to 0 ms) both for PRE and HYP trials, and then compare them to values computed during the (0–600 ms) intervals that include sounds. Only such a double-subtraction approach could enable us to determine, for instance, if the observed overall increase of gamma power during HYP as compared to PRE (last raw of Figure 5A) corresponded either (i) to a larger increase of gamma power elicited by sounds as compared to baseline during HYP condition than during PRE condition, or (ii) to a smaller decrease of gamma power elicited by sounds as compared to baseline during HYP condition than during PRE condition. The small duration of baseline windows (200 ms) prevented us to compute spectral power optimally in particular for slow frequencies (delta and theta values). Such analyses would allow to compare our findings to the previously reported effects in other works exploring spectral power correlates of auditory perception (Fontolan et al., 2014).

Likewise, we found a significant modulation of cortico-cortical functional connectivity measured by the scalp wSMI during hypnotic deafness. Interestingly the wSMI theta that discriminates conscious states from unconscious states in various conditions (King et al., 2013) did not differ between PRE and HYP that both correspond to conscious states (absence of any significant cluster and BF topography mostly in favor of the null hypothesis with a moderate level of evidence). We observed an increase of wSMI delta over fronto-central electrodes during HYP as compared to PRE (*p*-value = 0.0001, BF10 > 30 for most electrodes in the cluster), whereas an opposite pattern was present in the wSMI alpha, with a topography spreading from central to right posterior electrodes (*p*-value = 0.0019, BF10 > 30 for most electrodes in the cluster).

## Discussion

### Brief Summary of Our Main Findings

In this article, we present a case-study of hypnotic suggested deafness to elementary sounds in a highly suggestible healthy participant. Using an auditory odd-ball paradigm, we showed that while early evoked components where preserved, hypnotic deafness specifically abolished the late P3b component. Moreover, hypnotic deafness was associated with a centro-anterior midline positivity occurring within the time-window of the P3a component, with source localization suggesting a medial prefrontal activation and most specifically a dACC activation. Multivariate pattern analysis revealed two different profiles for baseline and hypnotic deafness conditions: while a trained classifier was able to differentiate deviant from standard trials during PRE condition – with a statistically significant performance against chance on the time window of the P300 component – classification was at chance-level during hypnotic deafness. A more sophisticated multivariate pattern analysis-the temporal generalization method-revealed a specific abolishment of the late and sustained pattern of decoding, previously associated with conscious access, during the hypnotic deafness condition. Finally, state markers calculated during the time-window of the first four sounds (“pseudo-resting state”) revealed significant differences between PRE and HYP conditions, that will be discussed below.

### Limitations of Our Study

Before further discussing our results, it is important to stress that our study had limitations. First of all, it is a case-study with only one participant, that we intended as a proof of concept. Therefore, we cannot guarantee that these results could generalize to a larger group of suggestible participants. Nevertheless, as we will discuss below, some of our results are compatible with finding of other studies in larger groups of hypnotically suggested perception deficits (Barabasz et al., 1999; Schmidt et al., 2017; Franz et al., 2020). More importantly, as the data of our third and last block (“POST” condition) was not analyzable, there is a potential time-factor confound in our results, as well as other potential mundane confound factors (for example, drying of electrodes during the HYP block that could cause an introduction of noise in the data). While this remains a major limitation of our study, it is important to note that: (i) the preservation of early processing contrasting with a massive difference of P3 components, in close accordance with subjective reports, makes it difficult to explain our dataset by this factors; (ii) our automatic rejection of artifact procedure usually guarantees that the remaining data is of very high quality, which should prevent mundane confound factors such as cited before (visual inspection of randomly selected portions of data in both conditions confirmed the quality of the remaining trials); (iii) finally, the fact that a multivariate classifier trained with HYP data was able to correctly classify PRE trials (deviant vs. standard), is an additional argument of the fact that the quality of the data was comparable between the two conditions.

### Preservation of Early Processing During Hypnotic Deafness

Our first main finding was that hypnotic deafness did not alter in a significant manner early cortical processing of sounds. Indeed, primary cortical responses to sounds (ex: P1) and even modular automatic responses to novelty MMN were not statistically different between PRE and hypnotic deafness (HYP), and bayesian analyses were in favor of the absence of difference between the two conditions, particularly for the MMN. Note, however, that a statistical trend was observed for a main effect of the state (HYP vs. PRE) for the P1 (evoked response of the first sound, *p*-value = 0.063, with Bayesian analysis not contributive with a BF10 = 0.69). If confirmed by future studies on a larger group of subjects, this trend could be accounted for by previously reported early effects of endogenous attention in vision and audition (Luck et al., 2000; O’Connor et al., 2002) combined with the reverse hierarchy theory of perception (Ahissar and Hochstein, 2004) which postulates that top-down effects (i.e., such as the one at stake in our study) would follow a gradient with larger effects on high-level cognitive processes than on low-level perceptual stages. Overall, our finding of a large preservation of early processing suggests a predominant late stage effect of hypnotic deafness that would specifically target conscious access stage as illustrated by the disappearance of P3b component in parallel to the conscious deafness reported by the volunteer.

### Hypnotic Deafness Is Associated With an Absence of a P3b

The disappearance of the P3b during the hypnotic deafness condition deserves several commentaries in the light of current knowledge related to neural signatures of conscious access.

First, this observation strengthens the theoretical proposal of the P3b as the neural signature of conscious access to sensory representations (Sergent et al., 2005; Del Cul et al., 2007; Gaillard et al., 2009; Naccache et al., 2016). It is noteworthy to mention here again that the volunteer was not engaged in an active counting task but simply in a “passive attentive” condition. Indeed, several authors proposed that rather than being a pure signature of conscious access, the P3b may rather index post-perceptual processes occurring later in time and related to the task being performed on consciously accessed stimuli (Aru et al., 2012; Tsuchiya et al., 2015; Cohen et al., 2020). This discussion also refers to the rigorous definition of “consciousness”: is a conscious experience necessarily self-reported or self-reportable, as postulated by several theories and models (Dehaene and Naccache, 2001; Dehaene et al., 2006; Lau and Rosenthal, 2011; Naccache, 2018) or is there a place for conscious but non-self-reported experiences (Zeki, 2003; Lamme, 2006; Block, 2007; Solms, 2021)? While this discussion is out of the scope of the present report, a recent empirical finding by Sergent et al. (2021) may suggest a solution: these authors took advantage of inter-trial variability to identify the brain dynamics associated with the processing of auditory stimuli presented close to conscious threshold, in an active (reporting) condition but also in a passive (no-report) condition. This approach allowed them to discover that, even in the absence of any task or behavior (passive no-report condition), the high-density electroencephalographic response to auditory stimuli shows bifurcation dynamics around 250–300 ms post-stimulus. The same stimulus gives rise to late sustained activity on some trials, and not on others, and this late neural activity is predictive of conscious reportability during the active condition. Crucially, source localization suggested that “task-free conscious access recruits the same neural networks as those associated with explicit report, except for frontal executive components,” such as Supplementary Motor Area. This distributed neural network included frontal (such as Inferior Prefrontal Cortex) and parietal structures, as predicted by global neuronal workspace theory and HOT theory. In other terms, this study suggests that when conscious access (and self-reportability) is dissociated from task and from external reports to an experimenter, the genuine neural signature of access does not appear as a canonical P3b, but still as a late fronto-parietal neural event. Within this context, we could use a similar approach on a larger dataset including several volunteers and including a post-hypnotic control condition. This objective is further motivated by our source localization results that suggest a full deactivation of frontal lobe structures during the P3b vanishing.

A second main question elicited by our P3b vanishing result concerns its mechanism and specificity to the hypnotic suggestion of deafness. In particular, given the absence of workable EEG data from the post-hypnotic control session, one could interpret this result as an attentional effect confound with time (see above) and independent from the hypnotic suggestion: the participant may have progressively allocated less endogenous attention to the stimuli considered as boring. In addition, this result could also be accounted for by a less specific effect of hypnosis, mainly a non-specific attentional modulation due to the hypnotic induction. However, while we cannot dismiss these alternative interpretations, several arguments make them unlikely. First, the volunteer clearly reported a full deafness to played sounds and not a simple distraction to sounds attributes (i.e., high vs. low tone; deviant vs. standard), nor a general state of inattention or drowsiness, and she was not engaged in an active deviant counting task, but simply in a “passive attentive” condition. Second, the appearance of a centro-frontal positivity, prior to P3b time-window, in the HYP condition is not easily accountable by any of the two other mentioned alternative hypotheses (see below).

### Hypnotic Deafness Is Associated With a Mesio-Frontal P3a-Like Component

The appearance of a specific fronto-mesial P3a like event associated with the disappearance of the P3b component is interesting for the following three reasons.

First, this EEG event is difficult to account for by a pure time-confound factor discussed above. In other terms, the appearance of this P3a suggests that the disappearance of the P3b cannot be easily explained by all possible factors confounded with time (i.e., attention, progressive increase of electrode impedances with drying,…).

Second, the co-occurrence of P3a appearance with P3b disappearance suggests a possible mechanism of conscious access inhibition setting. The possible dACC origin of this event further increases this interpretation by linking this region with the rich literature about cognitive inhibitory processes and executive control (Bush et al., 2000; Botvinick et al., 2001; Miller and Cohen, 2001). If confirmed by future studies, this specific inhibition mechanism would also suggest that the hypnotic intervention we applied here did not correspond to a general modification of state, irrespective of conscious contents, but rather to a targeted effect on access to such specific content as auditory tones. A simple and elegant way to confirm this proposal could consist of crossing two sensory modalities (e.g., vision and audition), and to induce a specific hypnosis-suggested inhibition of conscious access restricted to only one of them (e.g., crossing deafness with blindness). We would predict the presence of an anomalous P3a-like event, associated with an absence of the P3b, exclusively for the sensory modality inhibited by hypnosis, whereas a normal ERP profile would be observed for the other modality.

Third, this finding also links our study with previous reports suggesting an inhibitory role of ACC in hypnotic analgesia, by preventing conscious access to nociceptive representations (Faymonville et al., 2000; Trujillo-Rodríguez et al., 2019). Interestingly, while most other studies used PET or fMRI that cannot probe the fine dynamics of brain activity, our result suggest an early inhibition preventing conscious access and further activation of emotional section of the ACC such as the ventral ACC. In this respect, a replication of our finding with SEEG in epileptic patients could offer the optimal space and time resolution to confirm our hypothesis.

### Modification of Spectral and Functional Connectivity Markers During Hypnosis

We observed an overall power decrease of slow EEG activities in anterior frontal areas associated with a symmetric increase of beta and gamma activities. This spectral power finding further strengthens our hypothesis of an active (rather than passive) cognitive processing occurring during HYP session. Indeed, many studies associated fast activities with local cortical processing (Engel et al., 2001; Logothetis et al., 2001). Note also that this observed pattern is not easily explainable by the time confound factors mentioned above.

Functional connectivity measures revealed two additional findings. First, the conscious state marker (wSMI “theta”) – previously validated in conscious controls and in patients suffering from disorders of consciousness (King et al., 2013; Sitt et al., 2014; Bourdillon et al., 2019, 2020) – did not differ between PRE and HYP conditions. This negative finding supports the stability of conscious state during hypnosis, in opposition for instance with a decrease of vigilance and arousal that would have induced a decrease of the consciousness level. Second and more original, the decrease of wMSI in a faster frequency (wSMI “alpha”) is supporting the notion of functional disconnection of auditory areas from the global neuronal workspace. Indeed, many studies reported the role of alpha-beta range connectivity in conscious access to visual or auditory representations in cats (Gray and Singer, 1989) and in both non-human and human primates (Stein et al., 2000; von Stein and Sarnthein, 2000; Tallon-Baudry et al., 2001; Gross et al., 2004; Buschman and Miller, 2007; Gaillard et al., 2009).

### Relation to Other Studies of Hypnotic Induced Deafness

To the best of our knowledge, only two previous studies evaluated the effects of hypnotic induced deafness on brain responses to sounds using EEG and evoked response potentials (Barabasz et al., 1999; Franz et al., 2020). In the most recent one (Franz et al., 2020), a group of forty-eight participants (half of them being “highly suggestible” and the other half “low suggestible”) completed an auditory odd-ball paradigm during hypnotic “deafness” and during three other conditions (control, distraction, and simulation of hypnosis). In agreement with our own results, the authors found a preservation of early ERP components but a significant reduction of the P3b amplitude during hypnosis, as compared to control and distraction conditions. However, the results did not show the centro-anterior P3a-like component during hypnosis revealed in our own study. How can we account for this major difference? It is noteworthy that the specific suggestion delivered during the Franz et al. (2020) study was very different from ours: in their study, during hypnosis, participants were suggested that an earplug would obstruct the perception of tones. Crucially, in the study by Barabasz et al. (1999) the authors reported very different ERP profiles according to the specific suggestion that was delivered to the participants. More precisely, when the suggestion was that of an obstruction of the hearing of the tone pips due to virtual foam earplugs in their ears [as in the Franz et al. (2020) study], the results showed a reduction of the P300 component amplitude during hypnosis as compared to baseline condition, in highly suggestible individuals. By contrast, when the suggestion was that of a complete deafness to sounds (such as in the present report), the authors reported an increase in the P300 amplitude during hypnosis as compared to baseline in highly suggestible individuals. The Barabasz et al. (1999) study did not distinguish between early and late P300 components (respectively, P3a and P3b) neither in the temporal or spatial dimensions, and had in fact not enough spatial precision to distinguish the specific topographies of these two components (since the EEG montage relied on only five electrodes). Therefore, a plausible explanation is that, in the case of the specific suggestion of a complete deafness to sounds (“negative hallucination”), an anomalous and increased P3a-like response indeed exists (corresponding to an active inhibition process probably mediated by dACC as suggested before) as revealed in the present report, and explaining the apparent increase in the P300 component reported by Barabasz et al. (1999) due to a confound between this P3a-like component and the P3b (which is actually reduced or abolished). By opposition, the suggestion of an obstructive phenomenon (ex: virtual earplugs) resembles more to a “positive hallucination,” and possibly involves a different mechanism than an active inhibition process; therefore, only the abolition of the P3b is observed, without the P3a-like component, explaining the results of Franz et al. (2020). Of course, all of this remains hypothetical, and should be confirmed in future studies.

It is important to underline that our report is the first to our knowledge to complement classical ERP analysis with machine learning decoding methods and measures of state markers (PSD and connectivity measures) during an hypnotic induced deafness paradigm, providing a complete description of the brain dynamics during this condition.

### A Proposed Scenario of Hypnotic Deafness

As a conclusion, – and pending the several limitations mentioned above –, we propose the following mechanistic scenario for hypnotic deafness. Once the volunteer succeeds in adopting a conscious cognitive posture enabling hypnotic deafness to occur, here are the proposed neural stages of auditory stimuli.

(i) Early and unconscious processing of sounds in auditory areas and spanning from P1 to MMN is not affected by hypnotic induction;
(ii) Any auditory representation explicitly encoded in local auditory networks is prevented from accessing the GNW through an active inhibition mediated by dACC;
(iii) As a result, a transient functional disconnection of auditory areas from GNW prevents conscious access to this auditory representation;
(iv) While it is still possible to decode attributes of the auditory representation during the early stage, there is no trace of it in the GNW in the late period.

This GNW theory-based scenario may be tested, precised, and corrected in future experiments. More generally, this simple proposed scenario also illustrates the rich relevance of hypnotic suggestion to test specific cognitive and neural predictions related to conscious access, beyond the field of hypnosis *per se*.

## Data Availability Statement

The raw data supporting the conclusions of this article will be made available by the authors, without undue reservation.

## Ethics Statement

The studies involving human participants were reviewed and approved by the Ethical Committee of the Kremlin-Bicêtre Hospital (no. 98-25). The patients/participants provided their written informed consent to participate in this study.

## Author Contributions

EM: data analysis, production of the figures, interpretation of the results, and writing of the manuscript. BR: conception of the experimental design, recording of EEG data, and help with data analysis. AS: help with data analysis and proofreading of the manuscript. J-MB: evaluation of hypnotic suggestibility and hypnotic procedure during experiment (induction, suggestion of deafness, and termination of hypnosis). LN: conception of the experimental design, recording of EEG data, help with data analysis, interpretation of the results, and writing and correction of the manuscript. All authors contributed to the article and approved the submitted version.

## Conflict of Interest

The authors declare that the research was conducted in the absence of any commercial or financial relationships that could be construed as a potential conflict of interest.

## Publisher’s Note

All claims expressed in this article are solely those of the authors and do not necessarily represent those of their affiliated organizations, or those of the publisher, the editors and the reviewers. Any product that may be evaluated in this article, or claim that may be made by its manufacturer, is not guaranteed or endorsed by the publisher.

## Footnotes

1
https://neuroimage.usc.edu/brainstorm

2
https://jasp-stats.org/
